# Comparison of respiratory navigator techniques for interleaved high-resolution coronary vessel wall imaging

**DOI:** 10.1186/1532-429X-15-S1-E20

**Published:** 2013-01-30

**Authors:** Mehmet Akcakaya, Markus Henningsson, Reza Nezafat, Rene M Botnar

**Affiliations:** 1 Medicine, Beth Israel Deaconess Medical Center, Harvard Medical School, Boston, MA, USA; 2 Imaging Sciences & Biomedical Engineering, King's College London, London, UK

## Background

Three-dimensional high-resolution PSIR LGE [1], flow-independent vessel wall imaging [2], or T1 mapping [3] are all implemented with interleaved sequences, where the same volume is imaged multiple times with different inherent contrast. These volumes are then processed together to generate additional information, e.g. recovering the sign of magnetization, depicting vessel walls via subtraction, or calculating T1 maps via curve-fitting. Such techniques rely on correct registration of the voxels, hence on accurate motion compensation. In this study, we sought to explore the effects of synchronous and asynchronous navigator gating and tracking on respiratory motion compensation in high-resolution coronary vessel wall imaging with an interleaved T2prep sequence (i-T2prep).

## Methods

The right coronary artery (RCA) of 7 subjects (29.6±12.6 yrs) were imaged on a 1.5T Philips Achieva magnet with a targeted i-T2prep sequence using an interleaved SSFP acquisition (TR/TE/α=4.3/2.1 ms/90°, resolution=1×1×3 mm^3^, FOV=270×270×30 mm^3^) where one heartbeat (interleaf 1) was acquired with T2prep prepulse, and the subsequent one (interleaf 2) without [2]. A 5 mm gating window was used with the following strategies: 1) Accept the data only if both interleaves are within the gating window (synchronous), 2) Accept each interleaf independently (asynchronous). Acquisition time for each scan was recorded. Coronary vessel wall images were generated by weighted subtraction of the two interleaves [2], and quantitative vessel sharpness measurements were performed on these vessel wall images.

## Results

**Figure 1:**
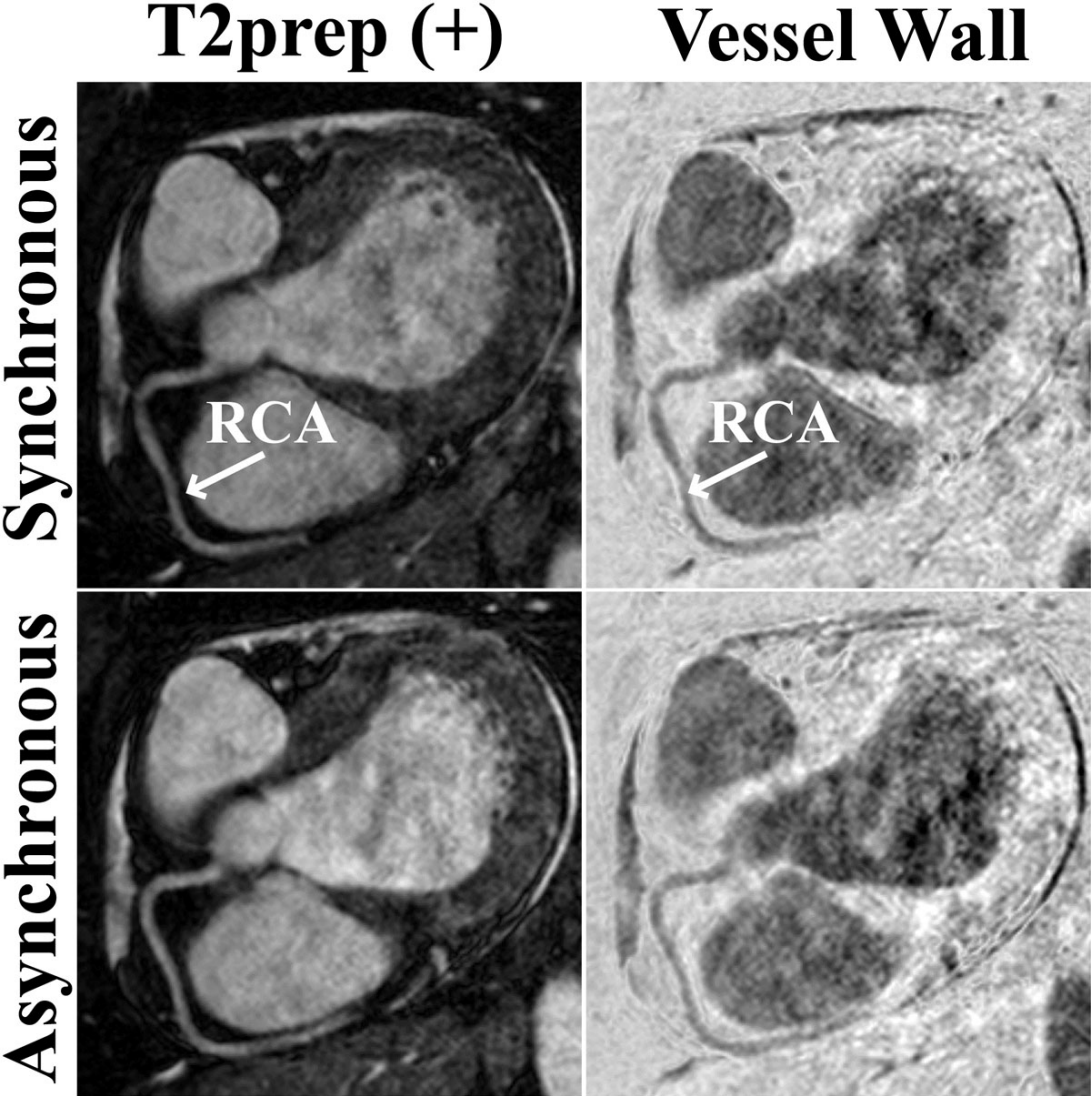
Reformatted images from targeted RCA acquisitions using an interleaved T2prep (i-T2prep) sequence with synchronous and asynchronous navigating techniques, depicting the interleaf with T2prep (T2prep(+)), and the coronary vessel wall image acquired via weighted subtraction of the two interleaves. Both navigating techniques yield images of similar quality. The asynchronous scheme results in a shorter acquisition (8:03 vs. 11:58 minutes).

**Figure 2:**
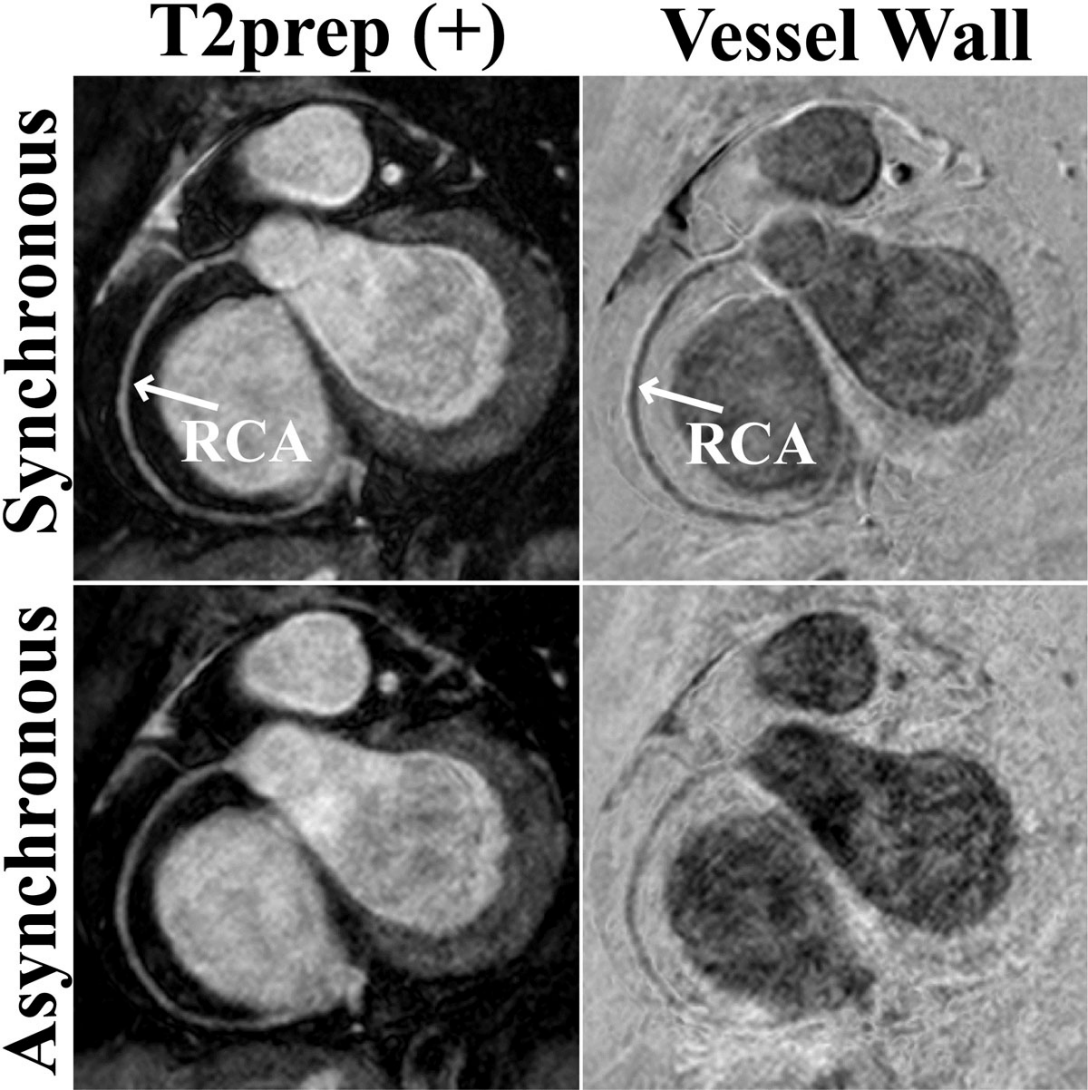
Reformatted images from targeted RCA acquisitions using an interleaved T2prep (i-T2prep) sequence with synchronous and asynchronous navigating techniques, depicting the interleaf with T2prep (T2prep(+)), and the coronary vessel wall image acquired via weighted subtraction of the two interleaves. While the T2prep(+) images are of similar quality, the synchronous technique yields a better depiction of the coronary vessel wall. The asynchronous technique is affected by motion within the 5mm gating window between the two interleaves. The synchronous scheme results in a longer acquisition (20:31 vs. 8:15 minutes).

Figure 1 shows reformatted RCA images with T2prep(+) and vessel wall images from a healthy subject. Both navigating techniques provide similar image quality, but the asynchronous scheme is shorter (8:03 vs. 11:58 mins). Figure 2 shows reformatted RCA images from another volunteer. The synchronous scheme has superior image quality, but also a longer acquisition time (20:31 vs. 8:15 mins). The synchronous scheme provides significantly better delineated vessel walls in terms of vessel sharpness (0.15±0.02 vs. 0.13±0.02, P=0.03) although the absolute difference is small. However, the asynchronous scheme is significantly shorter (9:29±1:35 vs. 18:18±6:21 mins, P=0.005).

## Conclusions

We have investigated two navigating approaches for high-resolution interleaved sequences. Examples and quantitative data show that while the asynchronous scheme allows a significantly shorter scan time, independently accepting data from interleaves results in loss of image quality and sharpness, even though a 5 mm gating window is utilized. Techniques that require voxel-by-voxel processing of images from multiple interleaves may require motion correction within the gating window if a synchronous scheme cannot be utilized due to a higher number of interleaves.

## Funding

NIH:K99HL111410-01;R01EB008743-01A2; BHF: RG/12/1/29262

## References

[B1] Kellman MRM 2003

[B2] Andia MRM 2012

[B3] Mehta JCMR 2012

